# Venetoclax for Acute Myeloid Leukemia in Pediatric Patients: A Texas Medical Center Experience

**DOI:** 10.3390/cancers15071983

**Published:** 2023-03-26

**Authors:** Adriana Trabal, Amber Gibson, Jiasen He, David McCall, Michael Roth, Cesar Nuñez, Miriam Garcia, Meredith Buzbee, Laurie Toepfer, Aram Bidikian, Naval Daver, Tapan Kadia, Nicholas J. Short, Ghayas C. Issa, Farhad Ravandi, Courtney D. DiNardo, Guillermo Montalban Bravo, Sofia Garces, Andrea Marcogliese, Hana Paek, Zoann Dreyer, Julienne Brackett, Michele Redell, Joanna Yi, Guillermo Garcia-Manero, Marina Konopleva, Alexandra Stevens, Branko Cuglievan

**Affiliations:** 1 Department of Pediatric Hematology/Oncology, Golisano Children’s Hospital, Fort Myers, FL 33908, USA; adriana.trabal@leehealth.org; 2 Department of Pediatric Oncology, The University of Texas MD Anderson Cancer Center, Houston, TX 77030, USA; algibson2@mdanderson.org (A.G.);; 3 Department of Leukemia, The University of Texas MD Anderson Cancer Center, Houston, TX 77030, USAggarciam@mdanderson.org (G.G.-M.); 4 Department of Pathology, The University of Texas MD Anderson Cancer Center, Houston, TX 77030, USA; 5 Department of Pathology & Immunology, Texas Children’s Hospital, Houston, TX 77030, USA; 6 Department of Pediatrics, Section of Hematology/Oncology, Texas Children’s Hospital, Baylor College of Medicine, Houston, TX 77030, USAamsteven@texaschildrens.org (A.S.); 7 Department of Oncology, Albert Einstein College of Medicine, Bronx, NY 10461, USA

**Keywords:** acute myeloid leukemia, pediatric, children, venetoclax, Bcl-2 inhibitor

## Abstract

**Simple Summary:**

Pediatric patients with relapsed or refractory acute myeloid leukemia (AML) have poor survival with current therapy. Venetoclax is a small molecule inhibitor that has shown promise in both adult and pediatric leukemias. Here we describe the joint experience of the Texas Medical Center (The University of Texas MD Anderson Cancer Center and Baylor College of Medicine/Texas Children’s Hospital) use of venetoclax in combination with various therapies for the treatment of pediatric relapsed AML. We report the safety and efficacy of this regimen in this population.

**Abstract:**

The BCL-2 inhibitor venetoclax improves survival for adult patients with acute myeloid leukemia (AML) in combination with lower-intensity therapies, but its benefit in pediatric patients with AML remains unclear. We retrospectively reviewed two Texas Medical Center institutions’ experience with venetoclax in 43 pediatric patients with AML; median age 17 years (range, 0.6–21). This population was highly refractory; 44% of patients (n = 19) had ≥3 prior lines of therapy, 37% (n = 16) had received a prior bone marrow transplant, and 81% (n = 35) had unfavorable genetics *KMT2A* (n = 17), WT1 (n = 13), FLT3-ITD (n = 10), monosomy 7 (n = 5), TP53 (n = 3), Inv(3) (n = 3), *IDH1/2* (n = 2), monosomy 5 (n = 1), *NUP98* (n = 1) and *ASXL1* (n = 1). The majority (86%) received venetoclax with a hypomethylating agent. Grade 3 or 4 adverse events included febrile neutropenia in 37% (n = 16), non-febrile neutropenia in 12% (n = 5), anemia in 14% (n = 6), and thrombocytopenia in 14% (n = 6). Of 40 patients evaluable for response, 10 patients (25%) achieved complete response (CR), 6 patients (15%) achieved CR with incomplete blood count recovery (CRi), and 2 patients (5%) had a partial response, (CR/CRi composite = 40%; ORR = 45%). Eleven (25%) patients received a hematopoietic stem cell transplant following venetoclax combination therapy, and six remain alive (median follow-up time 33.6 months). Median event-free survival and overall survival duration was 3.7 months and 8.7 months, respectively. Our findings suggest that in pediatric patients with AML, venetoclax is well-tolerated, with a safety profile similar to that in adults. More studies are needed to establish an optimal venetoclax-based regimen for the pediatric population.

## 1. Introduction

Pediatric acute myeloid leukemia (AML) is a rare hematopoietic neoplasm of the myeloid lineage. The estimated incidence in the United States is 7.7 per 1,000,000 children aged 1–14 years and increases with age [1]. Despite international collaborations that have advanced survival for these patients, the 5-year event-free survival (EFS) remains unacceptably low at 49% to 63%, with the most recent completed children’s oncology group (COG) trial reporting a 3-year EFS of 53.1% [2,3,4]. Approximately 30% of children with AML will subsequently develop bone marrow relapse with overall survival (OS) of less than 40% [5,6,7]. To date, there is no standard of care therapy for relapsed AML in pediatrics, though several novel agents such as menin inhibitors, CD123-directed therapies, venetoclax, and various FLT3 inhibitors are being actively studied [8].

Recently, the B-cell lymphoma 2 (BCL-2) protein has emerged as an exciting therapeutic target that has shown clinical benefits in adult patients with AML, both in the upfront and relapsed/refractory (r/r) setting [9,10,11]. BCL-2 is an important regulator of the apoptosis pathway. The overexpression of BCL-2 by AML and leukemia stem cells (LSC) has been implicated in accelerated disease progression and chemotherapy resistance [12,13]. Targeting this pathway with the BH3 mimetic, venetoclax, in combination with azacitidine (NCT02993523), proved so effective that venetoclax, in combination with hypomethylating agents (HMAs), received accelerated FDA approval as frontline therapy for elderly and unfit older patients who cannot tolerate intensive chemotherapy [14].

Despite this compelling evidence in adult patients with AML, little is known about venetoclax use and efficacy in pediatric patients, though numerous trials are currently ongoing to assess the efficacy and tolerability of this agent in this population (NCT03194932, NCT04161885, NCT03941964, NCT02250937, NCT04029688, NCT04000698, NCT03236857, NCT03844048, and NCT03826992) [15,16]. Recently Karol et al. evaluated the safety of venetoclax with conventional chemotherapy in pediatric patients with relapsed or refractory AML, and the efficacy results were extremely favorable [17]. In this study, we retrospectively review the collective Texas Medical Center experience for children treated at MD Anderson Cancer Center and Texas Children’s Hospital. The safety and efficacy of venetoclax in combination with other regimens for pediatric patients with relapsed and refractory AML is described. In addition, favorable (RUNX1-RUNX1T1, CBFB-MYH11, NPM1, and CEBPA bZIP) and unfavorable (MECOM, DEK-NUP214, KMT2A, NUP98, FLT3/ITD, WT1, monosomy 7, monosomy 5, TP53) genetic markers are increasingly used to guide management, so were identified and described here.

## 2. Materials and Methods

### 2.1. Patient Selection

This retrospective study was approved by the Institutional Review Boards of the University of Texas MD Anderson Cancer Center and Baylor College of Medicine/Texas Children’s Hospital. We reviewed these institutions’ electronic medical records to identify patients aged ≤21 years who received one or more cycles of venetoclax for relapsed/refractory AML at either institution between October 2017 and October 2021. Pathologic diagnoses were based on the World Health Organization classification of myeloid neoplasms and acute leukemias [18]. Clinically relevant cytogenetic and molecular mutations were captured. For each patient, the venetoclax dose, venetoclax-containing regimen and number of venetoclax cycles were documented.

### 2.2. Response and Adverse Event Evaluation

Bone marrow aspirations and biopsies were performed at the end of each cycle. Response to venetoclax was evaluated according to the revised International Working Group response criteria for AML [19]. Complete remission (CR) was defined as the disappearance of all clinical and/or radiologic evidence of disease, in addition to absolute neutrophil count (ANC) ≥ 1.0 × 10^3^/L, platelet count ≥ 100 × 10^3^/L, and bone marrow differential with <5% blasts. CR without blood count recovery (CRi) was defined as patients who met all criteria for CR but had either residual neutropenia (absolute neutrophil count < 1.0 × 10^3^/L) or thrombocytopenia (platelet count < 100 × 10^3^/L). Patients who had partial remission (PR) had a neutrophil count ≥ 1.0 × 10^3^/L and platelet count ≥ 100 × 10^3^/L but a bone marrow differential showing a decrease of at least 50% in the percentage of blasts from 5 to 25%. No response (NR) was defined as patients who did not meet any of the above criteria for response. An institutional eight-color multiparameter flow cytometry with a sensitivity level of 10^−4^ was used to assess bone marrow aspirates for minimal residual disease (MRD), and patients in CR with undetectable MRD are designated MRD negative [20]. Overall response rate (ORR) was defined as achieving CR, CRi, or PR. Based on clinical documentation, adverse events (AEs) attributed to venetoclax were graded according to the Common Terminology Criteria for Adverse Events version 5.0 (REF). Outpatient therapy was defined as ≥1 month of therapy received by outpatients that was not interrupted, with an inpatient admission of ≥7 days.

### 2.3. Statistical Analysis

Descriptive statistics were used to report patient characteristics, efficacy, and toxicity data. Kaplan–Meier curves were used to estimate OS (defined from venetoclax start date to death or censored at last follow-up) and event-free survival (EFS, defined from venetoclax start date to date of treatment failure, disease progression, relapse, death, or censored at last follow up) [21]. In univariate analyses, variables (age, sex, *KMT2A* status, prior hematopoietic stem cell transplant (HSCT), adverse genetics, number of prior lines of therapy, and status of venetoclax in combination with HMA) were summarized as numbers with percentages for categorical variables and as medians with ranges for continuous variables, with relation to response, both as a detailed response (NR, PR, CRi, CR) and binary response (PR, CRi, or CR, versus NR). Association was assessed by Chi-square test, Fisher’s exact test, analysis of variance, or two-sample *t*-test, as appropriate. To eliminate the impact of confounding variables, factors that had a *p*-value of ≤ 0.2 in univariate analysis were included in a multivariable logistic regression to identify predictors of response to venetoclax. Statistical analysis was conducted using GraphPad Prism 9 and IBM SPSS Statistics 26.

## 3. Results

### 3.1. Patient Characteristics

Forty-three patients with relapsed or refractory AML were included in our study. Their baseline characteristics are presented in Table 1. The median follow-up time was 33.6 months (range, 0.23–53.05 months). The median age was 17 years (range, 0.6–21 years). Sixty percent of patients were male (n = 26), forty-two percent (n = 18) were Caucasian, and 42% (n = 18) were Hispanic. Forty-four percent (n = 19) of our patients receiving venetoclax had refractory disease. Among the relapsed patients, 19% (n = 8) had prior remissions of ≤6 months, 14% (n = 6) had remissions that lasted 7–12 months and 12% (n = 5) had a prior remission that was greater than 12 months; the exact duration of prior remission was unknown for 5 patients. Forty-four percent (n = 19) had received three or more lines of prior therapy, and 37% (n = 16) had received a prior bone marrow transplant.

Molecular and cytogenetics evaluations revealed unfavorable genetics in 81% (n = 35) of patients, including *KMT2A* rearrangements (n = 17), WT1 (n = 13), FLT3-ITD (n = 10), monosomy 7 (n = 5), TP53 (n = 3), Inv(3) (n = 3), *IDH1/2* (n = 2), monosomy 5 (n = 1), *NUP98* (n = 1) and *ASXL1* (n = 1). Twenty-eight percent (n = 18) of patients harbored more than one unfavorable genetic alteration. RAS pathway mutations were found in 9% (n = 4) of patients. Favorable genetic alterations were found in 19% (n = 8) of patients, including RUNX1-RUNX1T1 (n = 2), CEBPA bZIP (n = 1), RUNX1-RUNX1T1 and CEBPA bZIP (n = 1), and NPM1 (n = 4). Results are shown in Table 2.

### 3.2. Treatment

The treatment characteristics of each patient are presented in Table 2. The median venetoclax treatment duration was 14 days in a 28-day cycle (range, 7–28 days), and the median number of venetoclax-containing cycles was 2 (range, 1–9 cycles). All patients were treated as inpatients at the start of therapy and received venetoclax daily. None of the patients received venetoclax as monotherapy. Eighty-six percent (n = 37) of patients received venetoclax with hypomethylating agents (HMAs) (decitabine or azacitidine). Sixty-five percent (n = 24) of patients treated with HMA/ venetoclax combination received outpatient therapy of >1 month duration with a median outpatient treatment duration of 1.81 months (range 1–12.57 months). Other regimens included venetoclax with FLAG-IDA (fludarabine, cytarabine, granulocyte-colony stimulating factor, and idarubicin) and venetoclax with cladribine, cytarabine, and idarubicin, but these patients did not receive outpatient therapy. Depending on disease cytogenetics and molecular features, other regimens included targeted agents such as gemtuzumab, gilteritinib, sorafenib, or midostaurin. Standard venetoclax dose escalation was performed on days 1–4 of cycle one in the majority of patients to decrease the risk of tumor lysis syndrome (TLS). Adolescents and young adults were dosed at the maximum FDA-approved dose of venetoclax of 400 mg; younger patients received recommended doses based on BSA (mg/m^2^) as listed in Table 2. Pre-emptive venetoclax dose adjustments ranging from 30% to 75% were employed in those patients with concurrent use of CYP3A inhibitors (e.g., azole antifungals, voriconazole, Posaconazole) as described in the FDA approval label.

### 3.3. Safety Profile

AE grade ≥3 that occurred during venetoclax use are shown in Table 3. The most common AEs were hematological. Grade 3 or 4 hematologic AEs included febrile neutropenia in 16 patients (37%), non-febrile neutropenia in 5 (12%), anemia in 6 (14%), and thrombocytopenia in 6 (14%). There were no grade 5 AEs attributed to venetoclax; 2 patients died of infection in the setting of refractory leukemia. Three patients (7%) had laboratory evidence of TLS that resolved with hydration and allopurinol and did not require rasburicase administration or discontinuation of therapy. Five patients (12%) had evidence of grade 4 sepsis with proven bacteremia. Isolated organisms included *S. viridans*, *S. epidermidis*, *K. pneumoniae*, and *R. mucilaginosa*. One patient developed pneumonia; no patients developed typhlitis. No new safety signals were identified in our patient cohort.

### 3.4. Response

**Figure 1:**
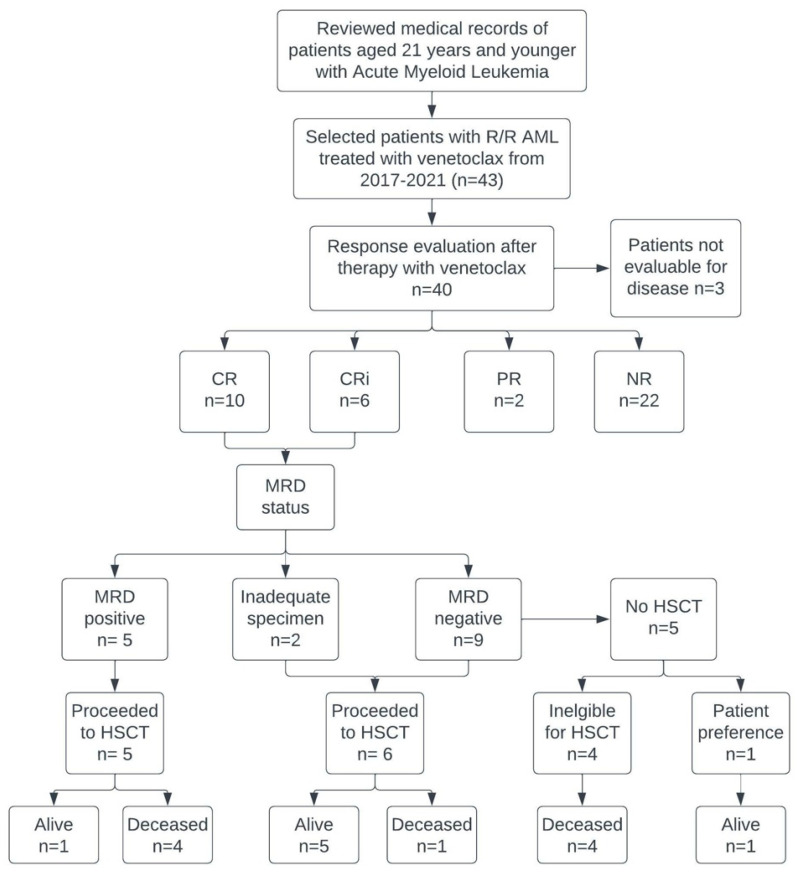
Flow chart for patient′s inclusion criteria and outcome. Flow chart for inclusion and overall outcome. Abbreviations: CR—complete response; CRi—complete response with incomplete blood count recovery; PR—partial response; NR—no response; HSCT—hematopoietic stem cell transplantation; MRD—measurable residual disease; R/R—relapsed refractory.

Three patients were not evaluable for response; two patients progressed and died before disease evaluation could be completed, and one patient was in CR prior to start of venetoclax which was used as a bridge to transplant (see Figure 1). Among the 40 evaluable patients, 25% (n = 10) had a CR, 15% (n = 6) achieved CRi, 5% (n = 2) achieved a PR, and 55% (n = 22) of patients had no response, yielding a composite CR/CRi of 40% and an ORR of 45%. Responses based on genetic alterations are summarized in Table 2. Favorable genetic alterations were identified in 8 patients and 50% (n = 4) of these achieved CR/CRi. Of patients with unfavorable genetic alterations 36% (n = 12) had CR/CRi. *KMT2A* rearrangement was the most common genetic alteration. Forty percent of patients (n = 6 of 15 evaluable) with *KMT2A* rearrangement had CR/CRi. Patients with WT1 mutations had a 33% CR/CRi rate. Twenty percent of patients with FLT3-ITD achieved CR/CRi. One of 3 patients with TP53 mutations achieved CR. There were 4 patients with NRAS and/or KRAS mutations and 75% (n = 3 of 4) achieved CR/CRi.

**Figure 2:**
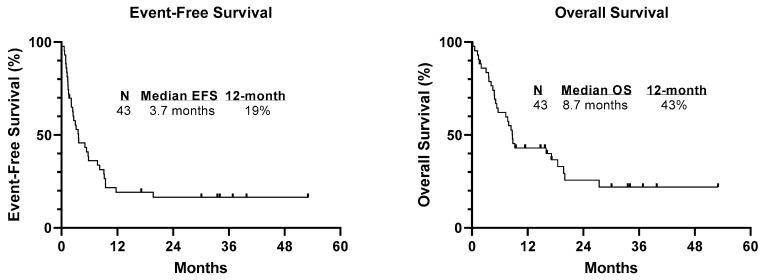
Survival curves. Legend: Kaplan–Meier curves showing EFS and OS from the start of venetoclax therapy.

Of the 16 patients who had a CR/CRi, 56% (n = 9) of patients were MRD negative after a median of 1 cycle of therapy, 5 patients had a CR but were MRD positive, and two patients were not evaluable for MRD due to limited sample availability. Thirteen patients had planned to proceed directly to HSCT in CR, but two relapsed before HSCT. Eleven patients (7 of whom received HMA/venetoclax) had a successful HSCT and of these, 6 remain alive with a median follow-up time of 33.6 months. Five patients did not receive HSCT when in remission after venetoclax therapy, 2 of these patients were deemed ineligible due to 2 prior HSCT’s and 2 of the patients progressed before getting to HSCT; all are now deceased from disease. One patient who was MRD negative chose not to pursue another HSCT and remains alive without disease with a follow-up time of 33.55 months. One patient received post-HSCT HMA/venetoclax for four cycles with tolerable side effects but ultimately progressed and pursued alternate therapy. The median event-free survival (EFS) and overall survival (OS) duration (see Figure 2) were 3.7 months (range 0.13–53.03 months) and 8.7 months (range 0.23–53.03 months), respectively.

### 3.5. Response with Venetoclax and Hypomethylating Agent

The majority of patients in our cohort were treated with HMA/venetoclax (86%, n = 37). Within this subset, 34 were evaluable for response assessment with an ORR of 44%. Of the 16 patients who achieved CR/CRi, 81% (n = 13) received HMA/venetoclax. Patients who received HMA and achieved CR proceeded to HSCT at a similar rate as those who were in CR from more myeloablative regimens such as FLAG-Ida-venetoclax or Cladribine (22% vs. 30%, respectively). Importantly, HMA/venetoclax allowed a large portion of patients to receive therapy as an outpatient regimen, with 65% (n = 24) receiving outpatient therapy of >1 month duration. In addition, two of the patients who met the definition of no response were able to achieve stable disease for >6 months each while receiving outpatient venetoclax combination therapies.

### 3.6. Prognostic Factors

Univariate and multivariate associations between response and each of the variables are summarized in Tables S1–S3. Patients who achieved response (CR/CRi/PR) with venetoclax-containing regimens had received a median of 2 prior lines of therapy (range 1–7) compared with a median of 3 lines of therapy in patients who did not achieve response (range 1–6, *p* = 0.1). In a multivariate analysis including age and the number of previous therapy lines, an increased number of prior lines of therapy was associated with lower odds of response to venetoclax (OR = 0.74), but this was not found to be statistically significant (95%CI: 0.46–1.17, *p* = 0.2).

## 4. Discussion

In this multi-institution retrospective study, we assessed the safety and efficacy of venetoclax in combination with standard chemotherapy, HMAs and/or tyrosine kinase inhibitor regimens in a heavily pretreated pediatric population where 44% of patients were being treated in the salvage-3 setting or beyond and 37% with a history of a prior bone marrow transplant. We found that venetoclax combination therapy is safe and well-tolerated in pediatric patients with r/r AML. Importantly, venetoclax in combination with HMA, provided a well-tolerated outpatient treatment option for the majority of patients. The ORR is comparable to that seen in heavily pretreated adult patients with AML who received similar venetoclax combination therapies [22]. Our data supports introducing venetoclax-containing regimens in cooperative group trials in pediatric patients with r/r AML.

In this cohort, the addition of venetoclax to a variety of regimens produced a tolerable AE profile. The most common AEs found were febrile neutropenia and bloodstream infections, which are consistent with other published data on venetoclax use in heavily pretreated pediatric patients with AML [15,17,23,24]. Karol et al. assessed the safety of venetoclax combined with chemotherapy in pediatric patients with relapsed or refractory AML, ref. [17] and reported that 66% of patients developed grade 3 or 4 febrile neutropenia. They reported that 16% developed invasive fungal infections and that 1 patient died from treatment-related colitis and sepsis. Although both studies had heavily pretreated patients with similar antimicrobial prophylaxis regimens, none of our patients developed fungal disease or died from treatment-related causes. This difference may be attributed to the longer duration of venetoclax (continuous for 28 days), the combination with a more myelosuppressive regimen including high dose cytarabine ± idarubicin or the use of prophylactic anti-fungal therapy [17]. The incidence of TLS in our study was low, similar to other AML trials that used this agent [14]. Ramp-up dosing and prior cytoreductive therapy for those with a high disease burden could have contributed to these results. All patients were medically managed and did not require intensive care for TLS.

Response assessment was limited since our population was highly heterogeneous from the clinical, molecular, and therapeutic standpoint. Nonetheless, several consistencies are worth highlighting. Our study included a substantial portion (86%, n = 37) of pediatric patients who were treated with venetoclax combined with a HMA, highlighting the fact that this is becoming a frequently used regimen for relapsed pediatric AML. The synergy between these two compounds has been demonstrated extensively in preclinical and clinical studies. Azacitidine inhibits the prosurvival proteins MCL1 and BCL-XL, thereby increasing the dependence of leukemia cells on BCL2, which is directly targeted by venetoclax. In our study, the combination led to encouraging ORR rates for a heavily pretreated population. These rates were comparable to those demonstrated when venetoclax was used with HMA in adults with r/r AML, albeit inferior to responses achieved when venetoclax has been combined with myelosuppressive agents such as FLAG-IDA [10,14,22,25,26]. Nevertheless, the combination allowed some patients to go for a consolidative HSCT, though the relapse rate after HSCT was ~50%. This could reflect a need for more cycles of venetoclax-containing cycles prior to transplant to achieve a deeper remission or a more targeted approach to identify mutational profiles which may convey increased sensitivity/resistance to venetoclax [14,27,28,29]. The safety profile of HMA/venetoclax warrants prospective evaluation as post-transplant maintenance in children. Indeed, one patient in our cohort received this combination for four cycles, which was well tolerated. In the adult population, post-transplant maintenance therapy with HMA/venetoclax has been shown to be safe and effective at decreasing relapse in adult patients with a 2-year EFS reported as 84% in a small study, with larger prospective studies (NCT04128501) underway [30].

In this multiply relapsed patient population, the tolerability of HMA/venetoclax made it a beneficial treatment strategy for pediatric patients who could not receive highly myelosuppressive regimens, anthracyclines, or were in the process of palliation. In our cohort, 65% (n = 24) of patients were able to receive outpatient therapy of >1 month duration with a median outpatient treatment duration of 1.81 months (range 1–12.57 months). It is also notable that even when patients were not able to achieve CR, it was a well-tolerated palliative approach for patients who had already rapidly progressed through multiple lines of therapy. Overall, the combination allowed for treatment flexibility; it could be given for shorter or longer treatment courses, could be used as a bridge while awaiting transplant, and, as stated, could provide treatment in the outpatient setting, which is unusual in pediatric AML.

A second point to emphasize in this cohort are the chromosomal and molecular patterns that emerged. Chromosomal rearrangements involving *KMT2A* on chromosome band 11q23 were the most common recurrent cytogenetic abnormality identified, comprising 40% of patients. This high proportion could be related to a selection bias, given the well-known clinical benefit of venetoclax when used in patients with this translocation [31]. Alternatively, it could be related to the high rates of treatment resistance and relapse, across all ages, of the specific *KMT2A* translocations identified in our cohort [15,21,22,32,33,34]. EFS rates of 34–61% and OS of 44–64% have been reported in this subgroup of patients, although outcomes are markedly inferior for higher-risk translocations such as those with partners 10p11.2, 10p12, or 6q27 [34]. In our cohort of 17 relapsed patients with *KMT2A*, 40% (n = 6) of patients achieved CR/CRi after a median of 1 cycle of a venetoclax-containing regimen. Of the patients with *KMT2A* who did not respond to venetoclax regimens, 4 of 10 had concurrent mutation profiles that have been associated with poor prognosis or venetoclax resistance (ASXL1, FLT3-ITD, WT1, TP53).

Intriguing data in adult patients with AML has emerged on increased sensitivity patterns to venetoclax seen with particular mutational profiles such as *NPM1*, *IDH1/2*, and *TET2* [13,35]. Therefore, we explored responses amongst patients in our cohort with these mutational profiles. Six patients had mutation profiles, including *NPM1*, *IDH1/2*, or *TET2* mutations; of these patients, three achieved CR/CRi. This poor response could have been associated with concurrent FLT3-ITD mutations seen in three of the patients with NMP1, or as shown in Issa et al., could be due to the r/r nature of the disease in our cohort [36]. Additionally, there is data in the adult population of venetoclax resistance profiles, including FLT3, TP53, and RAS mutations [13,24]. In our study, there were 16 patients with these resistance mutation profiles who had a 33% CR/CRi rate. Further studies are needed to determine if these mutational profiles can guide how and when to incorporate venetoclax into the treatment schemas of the cooperative groups.

Our study must be viewed with several limitations in mind, including its retrospective nature, relatively small size, and the diversity of concurrent therapy used in conjunction with venetoclax. Furthermore, there was selection bias in the treatment choices, limiting comparisons with other studies’ findings. Despite these limitations, our study is the first to combine data from the Texas Medical Center’s largest cancer centers to report the use of venetoclax in pediatric patients with AML. Our findings indicate that venetoclax is safe, well-tolerated, and can be easily combined with numerous agents in pediatric patients with AML while still maintaining efficacy. Whether earlier use of venetoclax would yield a more significant survival benefit in this population remains unclear.

## 5. Conclusions

In conclusion, the findings of this retrospective review demonstrate that venetoclax should be considered as a part of salvage chemotherapy in pediatric patients with r/r AML and that venetoclax in combination with a hypomethylating agent provides a well-tolerated regimen for children who are not candidates for intensive cytotoxic chemotherapy. Its incorporation into frontline trials for pediatric patients with AML, as has been done with success in adult populations, still needs further investigation. Venetoclax’s efficacy as a second-line treatment strategy is still lacking and extrapolation of results need to take into account the relevant genetic differences between children and adult. Additional studies are needed to establish optimal venetoclax dosing, determine the optimal duration of venetoclax-based therapy, and collect long-term safety and pharmacokinetic data in children and adolescents.

## Figures and Tables

**Table 1:** Baseline patient demographics and characteristics.

Patient Characteristics	
Characteristic	No. of Patients (%)
**Median age, years (range)**	17 (0.6–21)
**Sex**	
Male	26 (60)
Female	17 (40)
**Race**	
Asian	3 (7)
African American	4 (9)
Hispanic	18 (42)
Caucasian	18 (42)
**Number of prior regimens**	
1	9 (21)
2	15 (35)
3	8 (19)
≥4	11 (26)
**Number of prior HSCT**	
0	26 (60)
1	11 (26)
≥2	6 (14)
**Genetics**	
*KMT2A*	17 (40)
FLT3-ITD	10 (23)
WT1	13 (30)
Monosomy 7	5 (12)
*NPM1*	4 (9)
RAS	4 (9)
TP53	3 (7)
RUNX1-RUNX1T1	3 (7)
Inv (3)	3 (7)
CEBPA	2 (5)
*IDH1/2*	2 (5)
5q-	1 (2)
*ASXL1*	1 (2)
*NUP98*	1 (2)
Inv (16)	0 (0)

Baseline patient characteristics, number of prior regimens and prior hematopoietic stem cell transplants, and genetic and molecular alterations. Abbreviations: HSCT—hematopoietic stem cell transplant.

**Table 2:** Patient disease characteristics, concurrent therapy, dosing for venetoclax, number of cycles given, response, toxicity, and HSCT status.

Patient	Age/Sex	Cytogenetics	NGS and PCR	Prior Therapy Number	Prior HSCT	Venetoclax Regimen	Dosing mg/m^2^	Cycles	Best Response	Toxicity	Directly to HSCT
1	0.66/M	*KMT2A*	PTPN11	2	No	(1) ARAC/VEN; (2) AZA/VEN	66	2	NR	None	No
2	1/F	t(8;12), FLT3-ITD	Negative	3	No	AZA/VEN	93	1	NR	None	No
3	2/M	*KMT2A*	BCR-ABL	2	Yes (2)	AZA/VEN	83	1	CR	TLS	No
4	2/M	*KMT2A*	Negative	5	Yes (2)	AZA/VEN	85	3	CR	None	No
5	3/F	KDM5A	Negative	2	No	AZA/VEN	342	2	CR	Thrombocytopenia, Anemia	Yes
6	4/M	*KMT2A*	Negative	4	Yes (2)	DEC/VEN	479	1	NE	TLS, Sepsis	No
7	5/M	Complex karyotype	PTPN11	1	Yes (2)	(1) AZA/VEN/ trametinib; (2) AZA/VEN	93	3	PR	None	No
8	6/F	*KMT2A*	*ASXL1*	5	No	(1) DEC/VEN; (2) AZA/VEN	322	2	NR	Pancytopenia, Pneumonia	No
9	7/F	RUNX1-RUNX1T1, Inversion 8	Negative	2	No	AZA/VEN	83	1	CR	None	No
10	9/M	*KMT2A*	Negative	2	Yes	AZA/VEN	163	1	NR	Diarrhea	No
11	10/M	*KMT2A*, FLT3-ITD	STAG2	1	No	AZA/VEN/GO/TKI	247	1	NR	Hemolytic anemia	Yes
12	11/F	*KMT2A*	WT1	3	No	AZA/VEN	237	2	NR	Pancytopenia	No
13	14/M	*KMT2A*	WT1, EED, PHF6, CSF3R	1	No	AZA/VEN	227	2	CR	None	Yes
14	14/F	Del 5q	TP53	1	No	AZA/VEN	64	2	NR	None	No
15	14/M	FLT3-ITD	WT1	2	Yes	AZA/VEN	61	1	NR	Pancytopenia	No
16	14/M	Negative	Negative	2	No	AZA, VEN	374	2	CR	None	No
17	15/M	FLT3-ITD, *NUP98-NSD1*	PTPN11, WT1	2	Yes	(1) AZA/VEN; (2) CLIA/GO/VEN; (3) CDK inhibitor/VEN	(1) 63 (2,3) 138	6	NR	TLS, thrombocytopenia, FNA	No
18	16/F	*KMT2A*	STAT5	3	No	AZA/VEN	75	2	PR	Pancytopenia, Sepsis	No
19	17/F	t(9;11), *KMT2A*	PRPF40B, WT1	2	No	CLIA/Ven/GO	153	1	CRi	FNA	Yes
20	17/M	FLT3-ITD, inversion 3, monosomy 7	CALR, CBL, PTPN11, STAT5A, WT1	2	No	(1) FLAG/GO/TKI/VEN; (2) Cladribine/ARAC/arsenic/ TKI/VEN	(1) 62; (2) 124	(1) 1 (2) 1	NR	FNA	No
21	17/M	*KMT2A*	PTPN11	4	Yes	AZA/VEN	56	4	NR	None	No
22	17/F	Negative	Negative	6	Yes (2)	AZA/VEN	263	1	NR	Neutropenia	No
23	18/M	Negative	IKZF1, NF1, PTPN11, DNMT3A	3	Yes	DEC/VEN	122	3	NR	FNA	No
24	18/M	*KMT2A*	Negative	3	Yes	DEC/VEN	67	1	NR	None	No
25	18/M	*KMT2A*	JAK1	1	No	AZA/VEN	167	1	NE	None	Yes
26	18/F	MECOM(EVI1), Inv 3, monosomy 7	CUX1, WT1, PTNP11	4	No	(1) FLAG-IDA/VEN (2) VEN/Mcl-1 inhibitor	(1) 41 (2) 117	(1) 1 (2) 1	NR	Elevated liver enzymes	No
27	19/M	MECOM(EVI1)r, Inv 3, monosomy 7	NRAS, WT1	2	No	DEC/ VEN	(1) 92 (2) 46	3	CRi	FNA	Yes
28	19/M	t (3;3), monosomy 7, FLT3-ITD	BCORL1, PTPN11	7	No	(1) FLAG, VEN/TKI (2) DEC/VEN/TKI (3) VEN/TKI (4) DEC/VEN/TKI (5) VEN/TKI	126	(1) 1 (2) 2 (3) 1 (4) 3 (5) 9	NR/stable disease	FNA, Nausea	No
29	20/F	NPM1, t(4;8), t(7;8)	BCORL1, NPM1, PTPN11, WT1	5	Yes	DEC/VEN	118	1	NE	None	No
30	20/F	Negative	WT1	4	No	DEC/ VEN	90	2	NR	None	No
31	20/F	Negative	NRAS, KRAS	3	No	AZA/VEN	50	4	CRi	FNA	Yes
32	20/M	Monosomy 7	KRAS	3	No	(1) DEC/VEN (2) CLIA/VEN/GO	(1) 93 (2) 47	(1) 9 (2) 1	CRi	FNA	Yes
33	20/M	*KMT2A*	SMC1A	1	Yes	FLAG-Ida/VEN	110	2	CRi	FNA	Yes
34	20/M	FLT3-ITD	IDH2; NPM1	2	Yes	DEC/VEN/GO	105	1	NR	None	No
35	20/F	*KMT2A*	Negative	2	Yes	AZA/VEN	75	2	CR	Klebsiella sepsis	No
36	21/M	IDH2	IDH2	1	No	FLAG-Ida/VEN	44	2	CR	FNA, Sepsis	Yes
37	21/F	FLT3-1868a	PIGA, WT1	5	Yes (2)	AZA/VEN/gilteritinib	60	1	NR	FNA	No
38	21/F	RUNX1-RUNX1T1;	Negative	2	No	DEC/GO/VEN	118	4	CR	FNA	No
39	21/M	RUNX1-RUNX1T1	CEBPA, KIT, STATSA	3	No	DEC/VEN	60	1	NR	Sepsis (R. Mucilaginosa)	No
40	21/F	FLT3-ITD, NPM1	NPM1, RUNX1, SH2B3, TP53, WT1	4	Yes	(1) DEC/VEN/TKI (2) AZA/VEN	(1) 134 (2) 268	(1) 1 (2) 3	CR	FNA	Yes
41	21/M	Negative	CEBPA, WT1	2	No	(1) ARAC/VEN (2) DEC/VEN	37	(1) 1 (2) 1	NR	FNA	No
42	21/M	*KMT2A*	KRAS, NRAS, BRINP3, TP53	1	No	DEC/VEN	52	1	NR	FNA	No
43	21/M	FLT3-ITD	NPM1, GATA2	1	No	CLIA, VEN	50	2	CRi	FNA (S. viridans)	Yes

Patient characteristics, including age, sex, cytogenetic/molecular alterations, number of prior cycles of therapy, prior HSCT status, dose of venetoclax, concurrent threapy given, response, toxicity, and status of HSCT post venetoclax combination therapy. Abbreviations: NGS—next-generation sequencing. PCR—polymerase chain reaction. HSCT—hematopoietic stem cell transplant. F—female. M—male. Chemotherapy: ARAC—cytarabine; GO—gemtuzumab ozogamicin; VEN—venetoclax; AZA—azacitidine; DEC—decitabine. Response: NR—no response; CR—complete remission; CRi—complete remission without blood count recovery; PR—partial response; NE—not evaluable. Toxicity: FNA—febrile neutropenia; TLS—tumor lysis syndrome.

**Table 3:** Adverse events attributable to venetoclax per CTCAE v5.0 (Ref).

Adverse Event, n (%)	Grade ≥ 3	Grade 3, n	Grade 4, n	Grade 5, n
Febrile neutropenia	16 (37)	16	0	0
Non-febrile neutropenia	5 (12)	1	4	0
Anemia	6 (14)	6	0	0
Thrombocytopenia	6 (14)	0	6	0
Sepsis	5 (12)	0	5	0
Tumor lysis syndrome	3 (7)	3	0	0
Nausea/vomiting	1 (2)	1	0	0
Elevated ALT/AST	1 (2)	1	0	0
Pneumonia	1 (2)	1	0	0

The number of adverse events, grade 3, 4, or 5 in patients undergoing venetoclax therapy. Adverse events were graded according to the Common Terminology Criteria for Adverse Events (CTCAE) v. Abbreviations: AST/ALT—aspartate aminotransaminase/alanine aminotransferase.

## Data Availability

The data presented in this study are available on request from the corresponding author. The data are not publicly available due to confidential patient information involvement.

## Supplementary Materials

The following supporting information can be downloaded at: https://www.mdpi.com/article/10.3390/cancers15071983/s1. Table S1: Univariate Analysis (CR vs. CRi vs. PR vs. NR). Table S2: Univariate Analysis (Response [CR+CRi+PR] vs. No response). Table S3: Multivariate Analysis (Response vs. No Response).
