# Supplementary material for: Venetoclax for Acute Myeloid Leukemia in Pediatric Patients: A Texas Medical Center Experience
Source: Cancers (Basel). 2023 Mar 26;15(7):1983. doi: 10.3390/cancers15071983 (PMC10093646; doi:10.3390/cancers15071983)
Supplement: Supplementary file 1 [file cancers-15-01983-s001.zip › cancers-2237743-supplementary.pdf]

Table S1. Univariate Analysis (CR vs CRi vs PR vs NR)

Variable	CR (N=10)	CRi (N=6)	PR (N=2)	NR (N=22)	*P*
**Median age (range)**	14 (1 – 20)	20 (17 – 21)	11 (5 – 16)	17 (1 – 21)	0.2
**N Female (%)**	5 (50%)	2 (33%)	1 (50%)	8 (36%)	0.7
**N *KMT2A*r (%)**	4 (40%)	2 (33%)	1 (50%)	9 (41%)	0.9
**N Adverse genetics (%)**	5 (50%)	4 (67%)	2 (100%)	16 (73%)	0.1
**Median prior lines of therapy (range)**	2 (1 – 5)	2 (1 – 3)	2 (1 – 2)	3 (1 – 7)	0.2
**N Prior HSCT (%)**	4 (40%)	1 (17%)	1 (50%)	8 (36%)	0.5
**N HMA + Ven (%)**	9 (90%)	3 (50%)	2 (100%)	16 (73%)	0.3

Table S2. Univariate Analysis (Response [CR+CRi+PR] vs No response)

Variable	Response (N=18)	No response (N=22)	*P*
**Median age (range)**	18 (1 – 21)	17 (1 – 21)	0.2
**N Female (%)**	8 (44%)	8 (36%)	0.8
**N *KMT2A*r (%)**	8 (44%)	8 (36%)	0.8
**N Adverse genetics (%)**	14 (78%)	13 (59%)	0.7
**Median prior lines of therapy (range)**	2 (1 – 5)	3 (1 – 7)	0.1
**N prior HSCT (%)**	6 (33%)	8 (36%)	0.8
**N HMA + Ven (%)**	14 (78%)	16 (73%)	0.7

To eliminate the impact of confounding variables, factors that had a *P* value of ≤ 0.2 in univariate analysis were included in a multivariable logistic regression (This only applied to binary response)

Table S3. Multivariate Analysis (Response vs No Response)

Variable	OR	95% CI	*P*
**Age**	1.03	0.93 – 1.13	0.6
**Prior lines of therapy**	0.74	0.46 – 1.17	0.2
